# A Rare Case of Gemella haemolysans Infection of Knee Arthroplasty

**DOI:** 10.7759/cureus.17073

**Published:** 2021-08-10

**Authors:** Kanchi Patell, Abdul Rahman Al Armashi, Francisco J Somoza-Cano, Keyvan Ravakhah, Julia Han

**Affiliations:** 1 Internal Medicine, St. Vincent’s Medical Center, Cleveland, USA; 2 Internal Medicine, St. Vincent Charity Medical Center, Cleveland, USA; 3 Infectious Diseases, St. Vincent's Medical Center, Cleveland, USA

**Keywords:** total knee arthroplasty, gemella haemolysans, vancomycin, tka, gram-positive cocci

## Abstract

Gemella haemolysans is a facultative, catalase-negative, anaerobic, gram-positive cocci. It is known to mostly cause endocarditis, meningitis, peritonitis, and cerebral abscesses. However, it is extremely rare for this organism to cause infections of an orthopedic nature, with only a single report of infection in total knee arthroplasty (TKA). We present a rare case of knee arthroplasty infection caused by G. haemolysans four years after an uncomplicated TKA procedure.

## Introduction

Gemella haemolysans is a facultative, catalase-negative, anaerobic, gram-positive cocci, and normal commensal of the upper respiratory tract and oral mucosa [1]. It is known to mostly cause endocarditis [2-6], meningitis [7,8], peritonitis [9,10], and cerebral abscesses [1]. It is extremely rare for this organism to cause infections of an orthopedic nature, with only one report of a hip infection [11], and a single report of infection in total knee arthroplasty (TKA) [12]. The most common organisms causing infection following TKA are Staphylococcus aureus and Staphylococcus epidermidis. We present a rare case of knee arthroplasty infection caused by G. haemolysans four years after an uncomplicated TKA procedure.

## Case presentation

**Figure 1:**
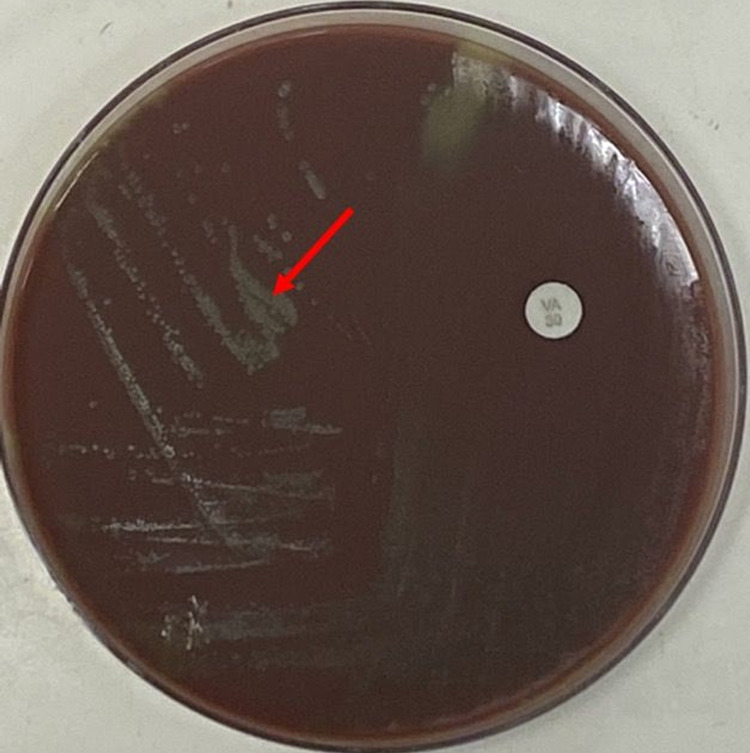
Alpha hemolysis on chocolate agar indicating G. haemolysans

**Figure 2:**
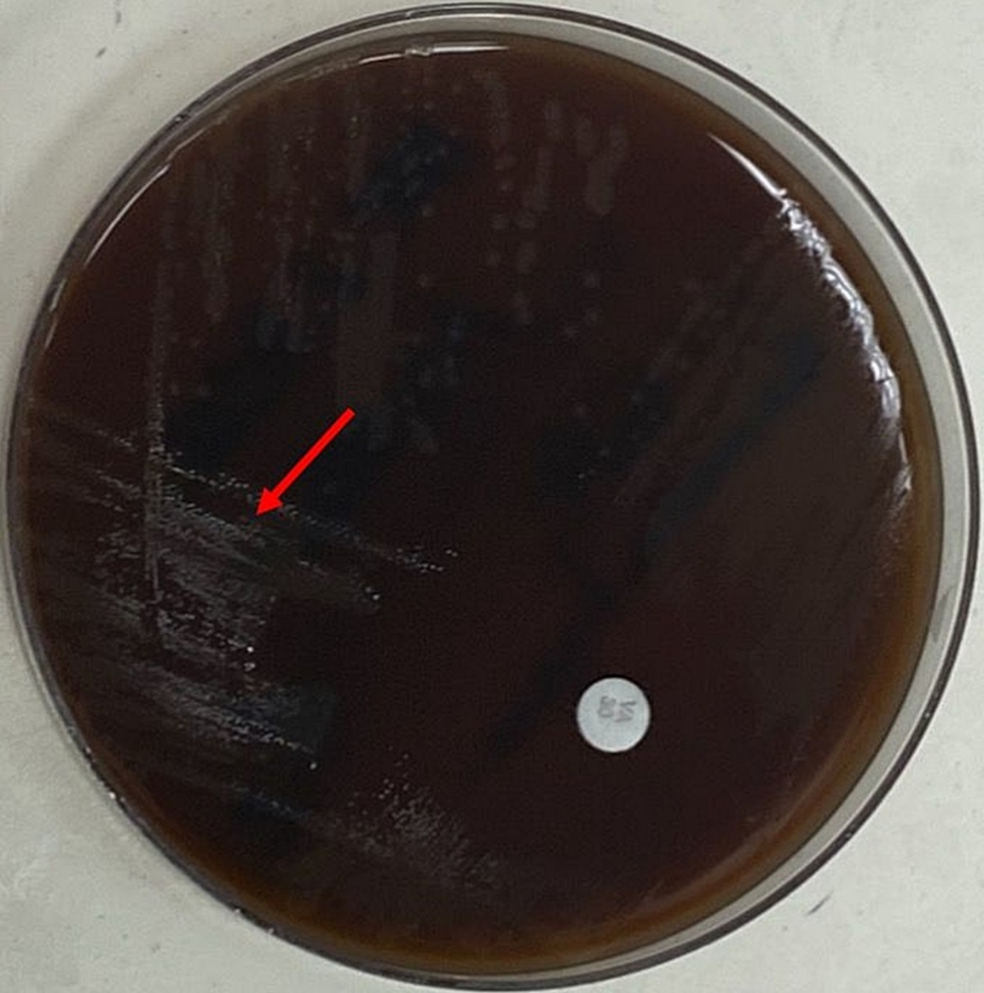
G. haemolysans on sheep blood agar

Our patient is a 64-year-old male with a past medical history including squamous cell carcinoma of the head and neck (diagnosed in 2004, status post resection and chemoradiation) and past surgical history of right TKA in 2018. He was admitted to the hospital complaining of right knee pain of seven-day duration. The patient started experiencing progressively worsening pain, swelling, and redness around his right knee for over one week. He denied any history of recent trauma, fever/chills, or other joint involvement. However, he stated undergoing routine scaling and root planing dental procedure two weeks before his symptoms started and was administered amoxicillin as a prophylaxis antibiotic. Vitals on admission showed temp: 36.5°C, blood pressure (BP): 166/77 mmHg, pulse: 74 bpm, and saturation of 95% on room air. Physical examination was significant for right knee erythema and edema. There was a considerably limited range of movement with marked tenderness on palpation and differential warmth around the right knee. Joint effusion was detected clinically. Knee arthrocentesis was performed, and the synovial fluid was sent for analysis and culture. Cardiovascular, respiratory, and abdominal examinations were normal. The white blood cell (WBC) count was 4,500/mL with a differential of 73.4% neutrophils, 16% lymphocytes, and 7.1% monocytes, ESR was 44 mm/hr, and C-reactive protein 78.2 mg/L. Synovial fluid analysis was turbid, fluid nucleated cells of 18,750/mm^3^, fluid neutrophils 91%, fluid lymphocytes 5%, and no fluid crystals were observed. Microbiological culture of the synovial fluid showed gram-positive cocci. Empirical treatment was initiated with vancomycin 1 g 12-hourly. Given the high probability of septic arthritis, the patient was taken to the operating room and right knee irrigation and debridement with polyethylene exchange were performed. The arthrocentesis synovial fluid culture 72 hours after collection showed alpha hemolysis on chocolate agar (Figure 1) and sheep blood agar (Figure 2), which was identified as G. haemolysans isolated from the subculture of thioglycolate broth.

 

Given the rarity of the organism, cultures were sent to Quest diagnostics in Valencia, California for sensitivity. The patient’s postoperative course was unremarkable with an improvement of knee edema and tenderness. He was making good progress towards all goals of physical therapy with an improved range of motion. He was discharged on daptomycin 6mg/kg 24-hourly for six weeks. Antibiotic susceptibility report for G. haemolysans came back after discharge and showed sensitivity for meropenem, ceftriaxone, clindamycin, penicillin, and vancomycin. Upon follow-up, he was doing well, and antibiotics were de-escalated to amoxicillin.

## Discussion

Gemella bacteremia is extremely rare. Six species have been classified as G. morbillorum, G. haemolysans, G. bergeri, G. sanguinis, G. palaticanis, and G. cuniculi using 16S rRNA gene sequencing [6,13]. Initially, G. haemolysans was described as Neisseria haemolysans [14] and was subsequently identified by Berger as G. haemolysans after biochemical differences were demonstrated with other Neisseria species [15]. G. haemolysans is usually an inhabitant bacterium of the upper respiratory tract or gastrointestinal tract [16]. It is an opportunistic pathogen reported to cause infection in immunocompromised patients, or those with underlying diabetes, alcohol abuse, or poor dental hygiene [17]. However, some cases have been reported in immunocompetent patients and have even caused life-threatening conditions in previously healthy people suggesting that the organism's pathogenicity should not be underestimated [18,19]. G. haemolysans are slow-growing and nutritionally fastidious bacteria. They are most likely to be isolated on rich, non-selective media such as blood or chocolate agar or thioglycolate broth [20]. They may exhibit alpha hemolysis on blood agar, hence resulting in initially being misidentified as viridans streptococci [21]. The cells are easily decolorized during gram staining and may, therefore, appear gram-variable or even gram-negative. This morphological polymorphism again leads to misidentification and explains why such few cases are reported [20,22]. G. haemolysans is thought to be sensitive to penicillin and ampicillin [23]. However, there is increased concern for the emergence of penicillin resistance in the Gemella species [13] and therefore a good treatment alternative is vancomycin, which even proved to be efficacious in our patient [2].

## Conclusions

Infections due to G. haemolysans are infrequent, let alone in relation to joints. To date, there is only one other case of infected TKA in a patient with rheumatoid arthritis who was successfully managed with two-stage revision surgery and penicillin. Our patient presented with infected knee arthroplasty four years after uncomplicated surgery. The most likely explanation for infection in our patient was the dental procedure he underwent two weeks prior to symptom onset in the setting of his underlying head and neck squamous cell carcinoma even though he was in remission. The best treatment for G. haemolysans prosthetic joint infection is unknown due to the limited case reports. So far, the case reports have managed patients with two-stage surgery. Our patient was managed with one-stage surgery followed by intravenous antibiotics and oral suppression. Despite the rarity of G. haemolysans, its pathogenicity should not be underestimated in both immunocompromised and immunocompetent patient populations.
